# Citicoline: A Cholinergic Precursor with a Pivotal Role in Dementia and Alzheimer’s Disease

**DOI:** 10.3233/JAD-240497

**Published:** 2024-07-16

**Authors:** Pietro Gareri, Antonino Maria Cotroneo, Roberta Montella, Matteo Gaglianone, Salvatore Putignano

**Affiliations:** a Unit of Frailty, Center of Cognitive Impairment and Dementia, Catanzaro Lido, ASP Catanzaro, Catanzaro Lido, Italy; b Director Complex Geriatric Unit, Maria Vittoria Hospital, Turin, Italy; c Medical Affairs, Piam Farmaceutici S.p.A, Genoa, Italy; d Geriatrician, Naples, Italy

**Keywords:** Alzheimer’s disease, citicoline, cognitive impairment, dementia, older patients

## Abstract

**Background:**

Citicoline is a naturally occurring compound with pleiotropic effects on neuronal function and cognitive processes.

**Objective:**

Based on previous studies, which shed light on the positive effects of citicoline 1 g when combined with acetylcholinesterase inhibitors (AChEIs) and/or memantine, we further investigated the benefits of citicoline in combination therapy in Alzheimer’s disease and mixed dementia.

**Methods:**

We integrated the datasets of CITIMEM and CITIDEMAGE, increasing the overall sample size to enhance statistical power. We analyzed data from these two investigator-initiated studies involving 295 patients. The primary outcome was the assessment over time of the effects of combined treatment versus memantine given alone or AChEI plus memantine on cognitive functions assessed by Mini-Mental State Examination (MMSE). The secondary outcomes were the influence of combined treatment on daily life functions, mood, and behavioral symptoms assessed by activities of daily life (ADL) and instrumental ADL, Geriatric Depression Scale, and Neuropsychiatric Inventory Scale. One-hundred-forty-three patients were treated with memantine and/or AChEI (control group), and 152 patients were treated with memantine and/or AChEI plus citicoline 1 g/day orally (Citicoline group).

**Results:**

A significant difference in MMSE score was found in the average between the two groups of treatment at 6 and 12 months.

**Conclusions:**

This study confirmed the effectiveness of combined citicoline treatment in patients with mixed dementia and Alzheimer’s disease, with a significant effect on the increase of MMSE score over time. The treated group also showed a significant reduction in the Geriatric Depression Scale and a significant increase in the instrumental ADL scale.

## INTRODUCTION

In the evolving landscape of cognitive research, the pursuit of effective interventions to ameliorate cognitive impairment remains a priority [1]. Cognitive decline poses a significant public health and social challenge issue with profound social implications, ranging from mild cognitive impairment to more severe conditions like Alzheimer’s disease (AD).

While current pharmacologic interventions may not offer a cure, they aim to alleviate symptoms and potentially slow down the progression of the disease. Among the approved drugs for AD are acetylcholinesterase inhibitors (AChEIs) and the N-methyl-D-aspartate (NMDA) receptor antagonist memantine, each targeting distinct pathways associated with the condition.

AChEIs, including rivastigmine, galantamine, and donepezil, constitute a common class of drugs used in AD treatment. These medications enhance cognitive functions by temporarily increasing acetylcholine levels in the brain. Indeed, they act on the brain acetylcholinesterase enzyme, promoting relative increases in acetylcholine abundance at the synaptic cleft for cholinergic neurotransmission. The efficacy of anticholinesterase inhibitors is similar among the individual drugs (donepezil, rivastigmine, galantamine). Despite AChEIs do not prevent neurodegeneration, they play a crucial role in managing cognitive symptoms associated with AD [2].

Memantine, an NMDA receptor antagonist, operates on a different pathological pathway by blocking receptors in the brain from excess stimulation that can lead to nerve cell damage. Therefore, memantine aims to protect neurons from further harm, offering a distinct approach compared to AChEIs [3–5]. Recognizing the limitations of individual drugs, the US Food and Drug Administration (FDA) approved a combination of memantine and donepezil, which aims to leverage the complementary and synergistic actions of both classes of drugs. The combination treatment with memantine and AChEIs was proven to be effective in treating AD [6–8].

The combination of different drugs reflects a strategic approach to address multiple pathological pathways associated with AD. This holistic approach reflects a continued effort to optimize treatment strategies, drawing on a growing body of evidence regarding the multifaceted benefits of combining different therapeutic agents in the management of cognitive decline associated with AD. Building upon this foundation, we researched the potential cognitive benefits of other cholinergic precursors, in particular citicoline, for its pleiotropic effects.

Citicoline is a naturally occurring compound, which is an essential intermediate in the synthesis of phosphatidylcholine and a crucial component of cell membranes, with potential benefits in Post Stroke Cognitive Impairment recovery and age-related cognitive decline [9]. Citicoline has emerged as a promising candidate, with preclinical and clinical studies suggesting a pleiotropic effect on neuronal function and cognitive processes [10, 11].

Among these studies, the CITIRIVAD and CITICHOLINAGE studies, conducted some years ago, shed light on the positive effects of citicoline 1 g when combined with AChEIs [12, 13]. More recently, we explored the cognitive impact of citicoline 1 g alongside memantine (CITIMEM) and the effectiveness of oral citicoline when administered in conjunction with AChEIs and memantine (CITIDEMAGE) [14, 15].

In this study, by integrating the datasets of CITIMEM and CITIDEMAGE, we increased the overall sample size to enhance statistical power. This approach may yield more robust and nuanced insights into the common underlying patterns or trends, uncovering interactions that might not be apparent when analyzing each dataset alone.

## METHODS

### Study population

We analyzed data obtained from two investigator-initiated studies involving a total of 295 patients. These studies are known as CITIMEM [14] and CITIDEMAGE [15].

The CITIMEM study, a retrospective analysis conducted between 2015 and 2017, included 126 patients (58 controls and 68 treated) aged 65 years or older, affected by mixed dementia (MD) and AD (mean age 80.7±5.2 years). Patients affected by AD or MD and treated with combination therapy or memantine were randomly selected within the 3-year timeframe [14].

The CITIDEMAGE study, also a retrospective case-control analysis, included 169 patients (85 controls and 84 treated) aged 65 years or older diagnosed with AD (mean age: 78.8±5.8 years). Patients affected by AD and treated with combination therapy (citicoline + memantine + an AChEI) or memantine + AChEI were randomly selected within a 3-year timeframe [15].

All participants had been enrolled after obtaining informed consent.

### Outcomes

• Primary outcome: effects of combined treatment versus memantine given alone or AChEI plus memantine on cognitive functions assessed by Mini-Mental State Examination (MMSE).
• Secondary outcomes: influence on daily life functions, mood, and behavioral symptoms assessed by activities of daily life (ADL) and IADL (instrumental ADL functions), Geriatric Depression Scale (GDS) for mood and Neuropsychiatric Inventory Scale (NPI) for behavioral symptoms.

### Tests

Cognitive functions were assessed using the MMSE [16, 17], daily life functions by ADL, IADL [18, 19], behavioral symptoms by NPI [20], and mood by GDS-short form [21].

• MMSE, a widely used dementia severity test, consists of 11 items with a total score ranging from 0 to 30. A lower score indicates greater impairment. The items encompass aspects of orientation, attention, short-term memory and short-term recall [16, 17].
• ADL covers essential self-care activities crucial for maintaining independence and overall well-being, such as bathing, dressing, toileting/continence, transferring/ambulating, and eating. A higher ADL score indicates greater independence, whereas a lower score suggests a higher degree of dependence, necessitating assistance or supervision [19, 22].
• IADLs refer to more complex activities related to independent living, including the use of the telephone and medication management [22]. A lower score indicates greater functional dependence [18].
• The NPI was utilized to assess the presence and severity of neuropsychiatric symptoms, with higher scores corresponding to more severe behavioral disorders [20].
• A 15-item GDS-Short Form is used for screening depression in the elderly population. A score of > 5 suggests depression [23].

Tests were administered at baseline (T0), after 6 (T1), and 12 months (T2).

For each endpoint, we studied its change value from the baseline. The baseline value was taken to be the value at visit 0 (0 months), and the changes were so calculated:

– change at time = 6 months: variable at T1 (at 6 months) – variable at T0 (baseline);
– change at time = 12 months: variable at T2 (at 12 months) – variable at T0 (baseline).

### Statistical analysis

Descriptive statistics will be provided for all variables in the summary tables by treatment group and study group according to the type of variable summarized. All the analyses were performed using the software R (version 4.3.1).

Quantitative variables will be summarized by using the number of subjects (n), number of missing values, arithmetic mean, standard deviation (SD), median, minimum, and maximum.

Categorical variables will be summarized by using frequency distributions and percentages.

Baseline values are defined as the Month 0 measurements (T0) unless otherwise specified for each variable.

Changes from baseline are calculated as the differences between the post-baseline value at each scheduled visit and the baseline value and will be summarized by treatment group reporting n, missing values, arithmetic mean, SD, median, minimum, and maximum. Change from baseline results was presented only when both baseline and post-baseline assessments were non-missing. The change in time for each endpoint was studied with a linear mixed model. When comparing means between treatment groups, we used the *t*-test; when the normality assumption of variable distribution was not satisfied, we used the Wilcoxon test.

Where provided, all confidence intervals will be two-sided 95% CIs and the significance level for all tests is taken to be 5%.

All the statistical analysis were done using the software R (version 4.3.1).

### Analysis sets

The analysis set includes all the patients who had already taken part in the CITIMEM and CITIDEMAGE studies. This means that this study involves 295 patients who had already been included in the single study satisfying all the inclusion criteria. The inclusion criteria were the same for both studies:

• Age of 65 years or older;
• People affected by AD or mixed dementia;
• People had to be on treatment with a single drug or combination therapy for at least 3 months from the scheduled start.

In particular:

• CITIMEM: 58 controls and 68 treated patients.
• CITIDEMAGE: 84 controls and 85 treated patients.

#### Univariate analyses

Subgroup categories will be created according to the definition below. Primary and key secondary efficacy endpoints will be analyzed by:

• Study (CITIDEMAGE/CITIMEM);
• Age group (≥65 years and < 76 years; ≥76 years and < 80 years; ≥80 years and < 83 years,≥83);
• Years of education group (≥1 year and < 5 years; ≥5 years and < 7 years; ≥7 years and < 8 years,≥8 years);
• Sex (male/female).

### Handling of missing and incomplete data

Interestingly, no imputation can be done for missing data on endpoints at their baseline value: Only data (at the baseline level for NPI) was missing, and that observation was not used in the model analysis.

### Software

All the analyses were conducted by using the software R version 4.3.1. Validation was done with Statistical Analysis Systems (SAS^®^) Software (release 9.4).

## RESULTS

### Disposition of subjects

Participants were previously examined in two individual studies [14, 15]. Baseline characteristics showed general similarities between groups, with the only differences observed at baseline being in MMSE and NPI scores, both of which were higher for the citicoline group, but not significant (*p* = 0.123).

A total of 143 patients were treated with memantine and/or AChEI (control group), and 152 patients were treated with memantine and/or AChEI plus citicoline 1 g/day orally (Citicoline group). Memantine dosage ranged from 10 to 20 mg/day in both groups, and donepezil dosage was 5– 10 mg. Rivastigmine patch dosage ranged from 9.5 mg to 13.3 mg, adjusted according to individual tolerability.

Table 1 reports the baseline characteristics of patients.

**Table 1:** Summary of baseline characteristics per treatment

Control	Citicoline	Total
(*n* = 143)	(*n* = 152)	(*n* = 295)
Age (y), mean (SD)	79.98 (6.01)	79.38 (5.26)	79.67 (5.64)
Sex, *n* (%)
Female	83 (58)	85 (56)	168 (57)
Male	60 (42)	67 (44)	127 (43)
Years of education in classes, *n* (%)
1–5	31 (22)	32 (21)	63 (21)
5–7	52 (36)	58 (38)	110 (37)
7–8	3 (2)	4 (3)	7 (2)
≥8	57 (40)	58 (38)	115 (39)
MMSE T0, mean (SD)	15.58 (2.95)	16.28 (2.89)	15.94 (2.93)
CIRS T0, mean (SD)	3.09 (1.06)	3.6 (1.04)	3.35 (1.08)
ADL T0, mean (SD)	3.24 (1.03)	3.34 (1.08)	3.29 (1.06)
IADL T0, mean (SD)	2.13 (1.12)	2.21 (1.25)	2.17 (1.19)
NPI T0, mean (SD)	11.56 (7.51)	13.03 (8.76)	12.32 (8.2)
GDS T0, mean (SD)	2.76 (1.34)	2.55 (1.33)	2.65 (1.34)

### Primary endpoint

#### MMSE

The mean change in MMSE from baseline was notably higher in the Citicoline group. When considering both the study and the timing of visits within the treated group, these factors significantly accounted for the observed increase in the mean change of MMSE.

**Fig. 1:**
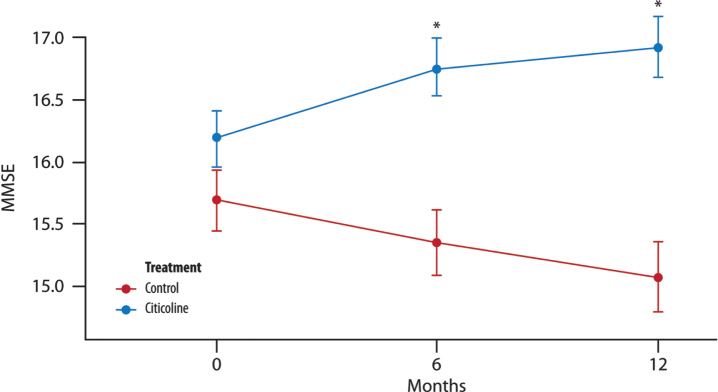
**The mean change in MMSE score over time**. Significant difference in the average between the two groups of treatment at each time point (T1, 6 months and T2, 12 months; *t*-test with a 5% significance level). ^*^p < 0.001 compared to controls (Wilcoxon-Rank Sum Tests to pairwise comparisons between group levels with Bonferroni’s corrections for multiple testing).

There was a significant difference between the two groups of treatment and also between the time points: the mean value for the change of MMSE from the baseline was smaller at 12 months than at 6 months (of about 0.28 units) (Fig. 1).

In the univariate analysis, we specifically noted significance in the model incorporating the interactions between years of education (grouped into classes) and treatment (both treated as factor variables). In this second model, both the coefficient for years of education and the interaction term (years of education * treatment) were found to be statisticallysignificant.

### Secondary endpoint

#### ADL

**Fig. 2:**
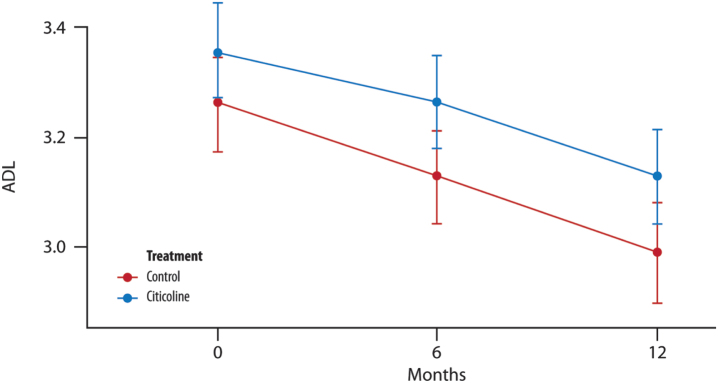
**The mean change in ADL score over time**. No significant difference in the average between the two groups of treatment at each time point (T1, 6 months and T2, 12 months) (*t*-test with a 5% significance level). *p* = 0.246 T1 versus treatment group; *p* = 0.277 T2 versus treatment group.

No changes were observed between the control and treated groups after 12 months of treatment (Fig. 2).

#### IADL

**Fig. 3:**
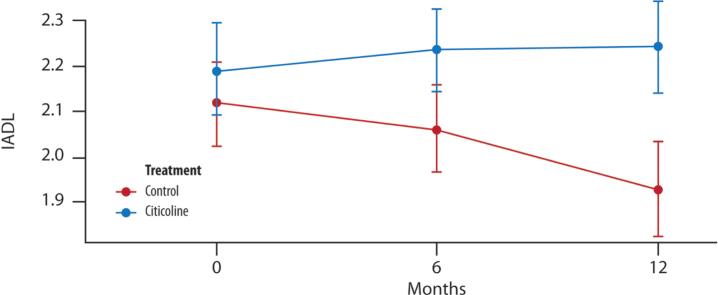
**The mean change in IADL score over time**. Difference in the average between the two groups of treatment at (T1, 6 months and T2, 12 months) (*t*-test with a 5% significance level). *p* = 0.189 T1 versus treatment group; ^*^p = 0.032 T2 versus treatment group.

A significant difference between the two treatment groups is evident only at the 12-month mark. The timing of visits, coupled with the treated group, significantly accounts for an increase in the mean change of IADL (Fig. 3).

Two models were used: initially, age was treated as a continuous variable, and the model selection based on the Akaike Information Criterion favored the inclusion of the age variable, as it proved to be significant. Subsequently, we considered age in classes, but during the stepwise search, it was not retained in the model.

In the univariate analysis, we also observed significance in the interaction term age*treatment.

#### NPI

**Fig. 4:**
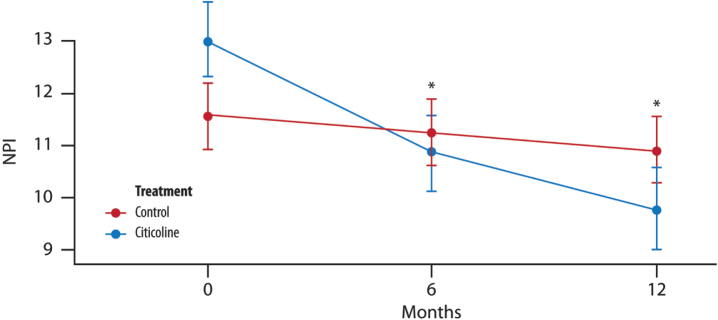
**The mean change in NPI score over time**. Difference in the average between the two groups of treatment at each time point (T1, 6 months and T2, 12 months) (*t*-test with a 5% significance level). *p* = 0.688 T1 versus treatment group; *p* = 0.275 T2 versus treatment group.

The mean change from baseline in NPI is lower for the treated group, and furthermore, both the study and the timing of visits significantly account for a decrease in the mean change of NPI (Fig. 4).

#### GDS

**Fig. 5:**
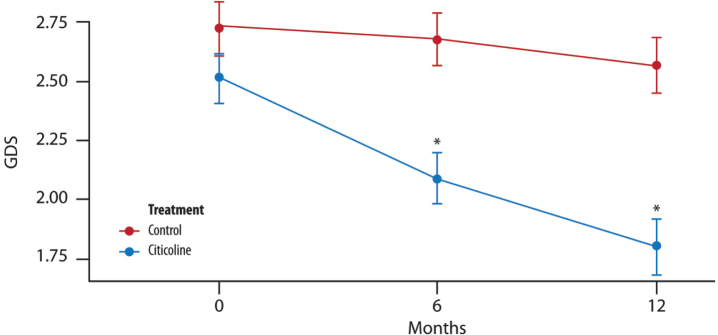
**The mean change in GDS score over time**. Significant difference in the average between the two groups of treatment at each time point (T1, 6 months and T2, 12 months) (*t*-test with a 5% significance level). ^*^p < 0.001 compared to control.

A significant difference in the mean is evident between the two treatment groups at 6 and 12 months (Fig. 5).

At each time point (T1, 6 months and T2, 12 months), there is a significant difference between the two groups of treatment (*t*-test with a 5% significance level).

Further data are reported in the Supplementary Material.

## DISCUSSION

The present study was definitely carried out on two investigator-initiated studies, the CITIMEM and the CITIDEMAGE Studies. It showed the benefits of using citicoline as an add-on treatment in AD and MD, together with memantine and/or AChEIs. The benefits can be appreciated in cognition, instrumental activities of daily living, and mood. In other words, it can give something more to the current usual treatments in AD and MD. Indeed, dementia is characterized by a chronic and progressive acquired loss of two or more cognitive abilities, including memory and independent functions [24, 25]. It is often accompanied by behavioral and psychological symptoms, such as agitation, depression, and apathy [24, 26].

Those who take care of dementia patients know how difficult managing dementia is. The overall goals are mainly focused on delaying the progressive cognitive decline and alleviating suffering caused by cognitive, behavioral, and psychological symptoms [24]. A well-designed dementia management plan can significantly enhance the quality of life for patients living with dementia and their caregivers [25].

Usually, therapy for cognitive symptoms in dementia patients begins with an acetylcholinesterase inhibitor or memantine [24, 25]. Furthermore, memantine and the AChEIs (donepezil, rivastigmine, and galantamine) can mitigate the progression of cognitive decline and functional loss in patients with AD, displaying a dose-dependent effect [13]. Memantine can also be used for patients who cannot tolerate an AChEI (for bradycardia or gastrointestinal diseases, such as nausea, vomiting, and diarrhea) [24]. A previous review indicated that the combination therapy of memantine and AChEI yields greater benefits in AD compared to AChEIs alone. However, the clinical relevance depends on the specific studies included [27]. On the other side, treatment with memantine and AChEIs is generally well tolerated; however, elevated doses of AChEIs are linked to more severe adverse events, such as vomiting and syncope. Overall, the combination of AChEIs and memantine has been shown to reduce the MMSE score by 1 point per year, similar to the observed impact of monoclonal antibodies. However, monoclonal antibodies are not economically sustainable and show some adverse events [28].

Along the same lines, the present study strengthens the evidence of the two previous studies of the combination treatment of citicoline with AChEIs and or memantine (CITIMEM and CITIDEMAGE studies). Indeed, we increased the overall sample size to enhance statistical power by integrating the datasets of CITIMEM and CITIDEMAGE, discovering interactions that were not apparent when analyzing each dataset alone.

The present study confirms the effectiveness of a prolonged combined citicoline treatment in patients with MD and AD (until 1 year). Specifically, after 12 months, the treatment with citicoline 1 g, along with memantine or AChEIs, demonstrated improvements in MMSE and IADL scores over time and significantly reduced GDS compared to treatment with memantine and/or AChEI alone.

The results align with previous research demonstrating that the combination treatment with memantine, AChEIs and citicoline appears to work together both in AD and mixed dementia [12–14, 29]. The optimal drug treatment may involve multiple drugs, each one with an effect size that may fall below the minimum clinically important difference [3].

In the present study, citicoline in add-on treatment with AChEIs and/or memantine improved the MMSE score by 2 points after 12 months of treatment, compared to the treatment with ACheIs and/or memantine alone.

Furthermore, citicoline also improved daily activities with an impact on the caregiver’s quality of life. Indeed, in the triple treatment group (citicoline group), there was a significant increase in the IADL score, and this is noteworthy as caregivers evaluate the smallest decline as clinically meaningful. This suggests that even a subtle decline in IADL functioning has a meaningful impact on the daily life of a patient [30]. Furthermore, there was a decrease in the NPI of 3.24 points in the citicoline group after 12 months of treatment. The NPI scale is considered the gold standard for neuropsychiatric symptoms common in dementia [31]. Neuropsychiatric symptoms have a big impact on a patient’s and his/her caregiver’s quality of life. The decrease in NPI score might be attributable to the improvement in mood, a common effect reported following the use of citicoline. Improvement in mood was also indicated by a slight decrease in the GDS score of 0.71 points in the citicoline group after 12 months. A slight change in scores (–0.15) was observed in the control group at the 12 months of treatment, and this is meaningful because of the importance of chronic administration.

The cognition and mood-enhancing effects of the triple therapy could be attributed to the synergistic action of citicoline, memantine and AChEIs; memantine plus AChEIs act on cognition, the add-on therapy with citicoline exerts a notable influence on cholinergic, noradrenergic, and dopaminergic neurotransmission, as well as the synthesis of serotonin via S-adenosyl-methionine [32, 33]. Furthermore, the add-on treatment with citicoline is particularly advantageous over an extended period (6–12 months), as it can increase acetylcholine levels and protect neuronal membranes [34–36]. Citicoline also has several remarkable actions:

• Prevents the accumulation of free fatty acids and the generation of free radicals at the site of ischemia [37–39].
• Decreases neuronal glutamate efflux and stimulates glutathione synthesis, a powerful antioxidant [40–43].
• Inhibits apoptosis and promotes mitochondrial energy metabolism by preventing the loss of cardiolipin [32, 33], thereby exerting neuroprotective effects and fostering synaptogenesis, neurogenesis, and gliogenesis [40–43].
• Improves the expression of SIRT-1, a neuroprotective protein able to activate the transcription of ADAM10 [44].
• Previous studies reported an involvement of dysfunctional microcerebral circulation in cognitive decline [45]. Indeed, CDP-choline influences cognitive and cerebrovascular function in AD, probably through a mechanism linked to an immunogenic and/or neurotrophic effect at the microvascular niche [45]. The use of transcranial Doppler could better stratify older patients with initial signs of cognitive impairment [46]. In future research, it would be interesting to investigate the effects of citicoline on microcerebral circulation through transcranial Doppler.

Citicoline treatment is also well tolerated [47]. Notably, choline in citicoline is less prone to conversion to trimethylamine, a gaseous metabolite readily taken up and oxidized in the liver to its atherogenic N-oxide tri-methylamine-N-oxide, compared to choline alphoscerate. This metabolite has been implicated in the etiology of different diseases, such as kidney failure, diabetes and cancer and an increased incidence of myocardial infarction, stroke or death and AD [48–50].

The present study has some limitations. Firstly, background therapies are different between the two studies; in the CITIMEM, only memantine was used, whereas, in the CITIDEMAGE study, AChEIs were added to memantine (the use of the Study variable should enclose this variability).

Additionally, baseline characteristics showed overall similarities between the groups, with the only notable differences observed at baseline being in MMSE scores, both of which were higher for the citicoline group. The disparity may be associated with the challenge of achieving homogeneity when combining data from two different studies.

### Conclusions

The treatment with citicoline was associated with positive benefits throughout the entire course of the disease, as reflected in both cognitive and functional outcomes. Citicoline, in add-on treatment with AChEIs and/or memantine, could help delay disease progression. Citicoline was found to have a significant effect on the increase of MMSE score over time. The treated group also showed a significant reduction in the GDS and a significant increase in the IADL. Further studies are necessary to confirm these results.

## AUTHOR CONTRIBUTIONS

Pietro Gareri (Conceptualization; Data curation; Formal analysis; Methodology; Resources; Writing – original draft; Writing – review & editing); Antonino Maria Cotroneo (Resources; Writing – review & editing); Roberta Montella (Conceptualization; Data curation; Formal analysis; Methodology; Writing – review & editing); Matteo Gaglianone (Conceptualization; Data curation; Methodology; Writing – review & editing); Salvatore Putignano (Resources; Writing – review & editing).

## Supplementary Material

Supplementary Material

## Data Availability

The data supporting the findings of this study are available on request from the corresponding author. The data are not publicly available due to privacy or ethical restrictions.
