# Supplementary material for: Citicoline: A Cholinergic Precursor with a Pivotal Role in Dementia and Alzheimer’s Disease
Source: J Alzheimers Dis. 2024 Jul 16;100(2):725–33. doi: 10.3233/JAD-240497 (PMC11307077; doi:10.3233/JAD-240497)

Supplementary Material

Citicoline: A Cholinergic Precursor with a Pivotal Role in Dementia and Alzheimer's Disease

Mini-Mental State Examination (MMSE)

Supplementary Table 1A. Summary statistics of MMSE with changes from baseline at each visit.

	Control, mean (SD)		Citicoline, mean (SD)		Total, mean (SD)	
	**Obs. value**	**Change**	**Obs. value**	**Change**	**Obs. value**	**Change**
**Baseline**	15.58 (2.95)		16.28 (2.89)		15.94 (2.93)	
**6 months**	15.24 (3.15)	-0.35 (0.96)	16.86 (2.93)	0.58 (0.99)	16.07 (3.14)	0.13 (1.08)
**12 months**	14.96 (3.36)	-0.63 (1.49)	17.03 (3.09)	0.75 (1.25)	16.02 (3.38)	0.08 (1.53)

As we can see from the Table, the treatment and the interaction between time and treatment have a significant effect in explaining the MMSE variable. Supplementary Table 1B will highlight the pairs that have led the interaction term to be significant at a level of 5%.

Supplementary Table 1B. Wilcoxon-Rank sum tests to pairwise comparisons between group levels with corrections for multiple testing (Bonferroni's correction).

	**T0: Control**	**T0: Citicoline**	**T1: Control**	**T1: Citicoline**	**T2: Control**
**T0: Citicoline**	1				
**T1: Control**	1	0.077			
**T1: Citicoline**	0.015	0.945	<0.001		
**T2: Control**	1	0.009	1	<0.001	
**T2: Citicoline**	0.003	0.282	<0.001	1	<0.001

Supplementary Figure 1. Two-way ANOVA with repeated measures over time (MMSE versus time and treatment). At each time point (indicated in the plot with T0, baseline, T1, 6 months and T2, 12 months), we found a significant difference in the average between the two groups of treatment (t-test with a 5% significance level).

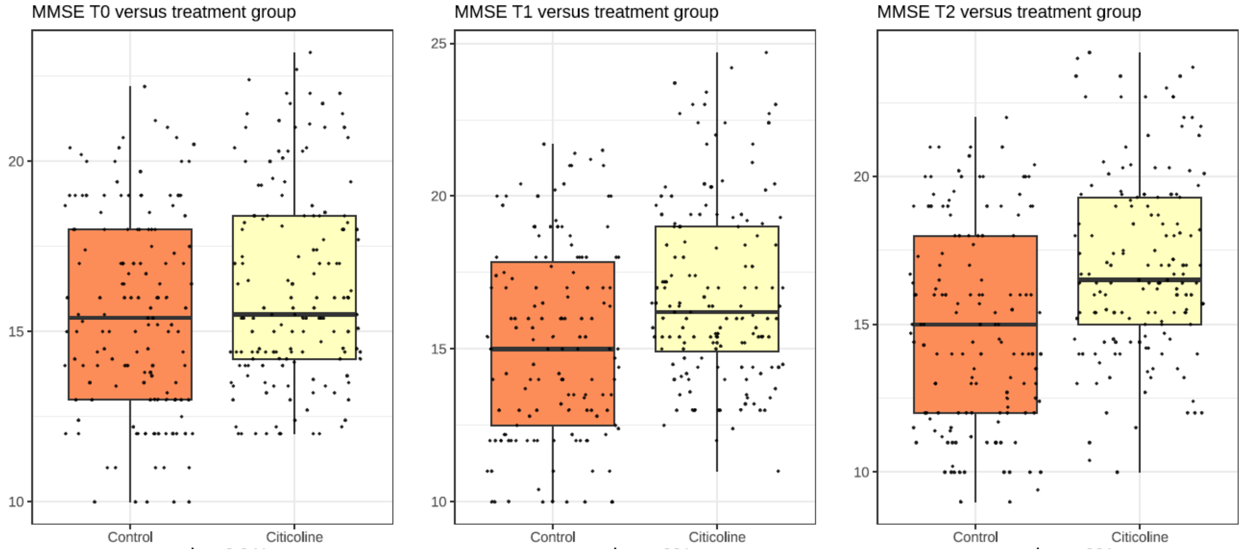

Activities of Daily Life (ADL)

Table 2. Summary statistics of ADL with changes from baseline at each visit.

	**Control, mean (SD)**		**Citicoline, mean (SD)**		**Total, mean (SD)**	
	**Obs. value**	**Change**	**Obs. value**	**Change**	**Obs. value**	**Change**
Baseline	3.24 (1.03)		3.34 (1.08)		3.29 (1.06)	
6 months	3.11 (1.01)	-0.13 (0.34)	3.25 (1.02)	-0.09 (0.51)	3.18 (1.02)	-0.11 (0.43)
12 months	2.97 (1.12)	-0.27 (0.6)	3.11 (1.08)	-0.23 (0.78)	3.04 (1.1)	-0.25 (0.69)

Figure 2. Two-way ANOVA with repeated measures over time (ADL versus time and treatment). At each time point (indicated in the plot with T0, baseline, T1, 6 months and T2, 12 months) we found a significant difference in mean between the two groups of treatment (t-test with a 5% significance level).

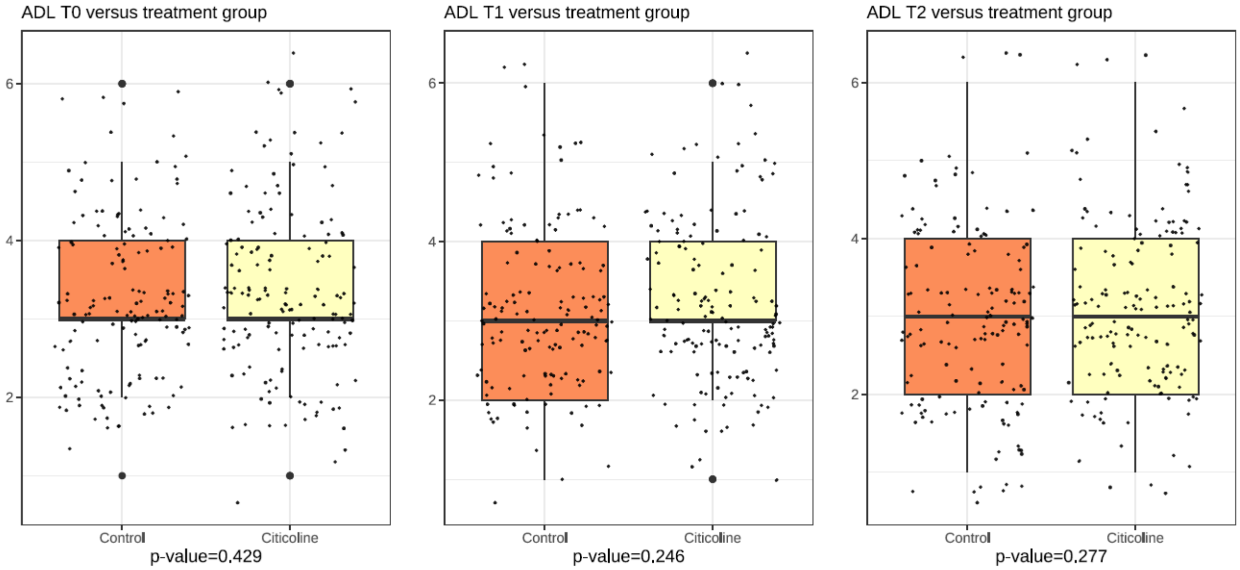

Instrumental Activities of Daily Life (IADL)

Table 3. Summary statistics of IADL with changes from baseline at each visit.

	Control, mean (SD)		Citicoline, mean (SD)		Total, mean (SD)	
	**Obs. value**	**Change**	**Obs. value**	**Change**	**Obs. value**	**Change**
Baseline	2.13 (1.12)		2.21 (1.25)		2.17 (1.19)	
6 months	2.08 (1.14)	-0.06 (0.23)	2.25 (1.11)	0.04 (0.61)	2.17 (1.13)	-0.01 (0.47)
12 months	1.94 (1.24)	-0.19 (0.43)	2.26 (1.26)	0.05 (1)	2.11 (1.26)	-0.07 (0.78)

Figure 3. Two-way ANOVA with repeated measures over time (IADL versus time and treatment). At each time point (indicated in the plot with T0, baseline, T1, 6 months and T2, 12 months), we found a significant difference in mean between the two groups of treatment (t-test with a 5% significance level).

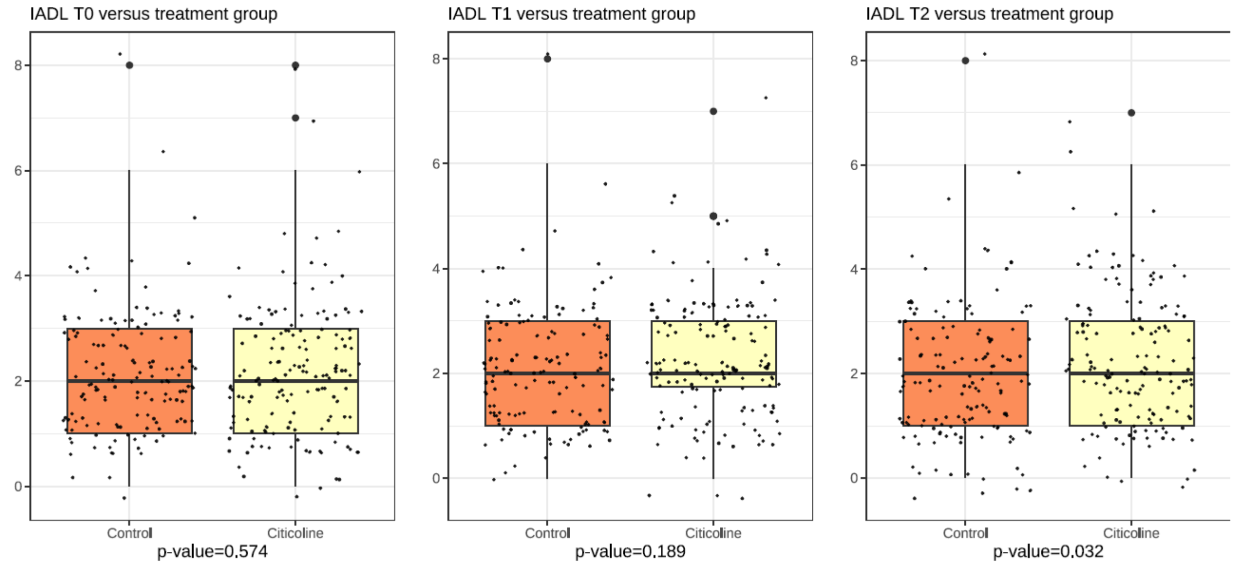

Neuropsychiatric Inventory Scale (NPI)

Table 4. Summary statistics of NPI with changes from baseline at each visit.

	Control, mean (SD)		Citicoline, mean (SD)		Total, mean (SD)	
	**Obs. value**	**Change**	**Obs. value**	**Change**	**Obs. value**	**Change**
Baseline	11.56 (7.51)		13.03 (8.76)		13.03 (8.76)	
6 months	11.25 (7.58)	-0.31 (1.18)	10.86 (9.06)	-2.17 (2.49)	11.04 (8.37)	-1.27 (2.18)
12 months	10.91 (7.55)	-0.65 (1.63)	9.79 (9.89)	-3.24 (4.18)	10.33 (8.85)	-1.99 (3.46)

Figure 4. At each time point (indicated in the plot with T0, baseline, T1, 6 months and T2, 12 months), there was a non-significant difference in mean between the two groups of treatment (t-test with a 5% significance level).

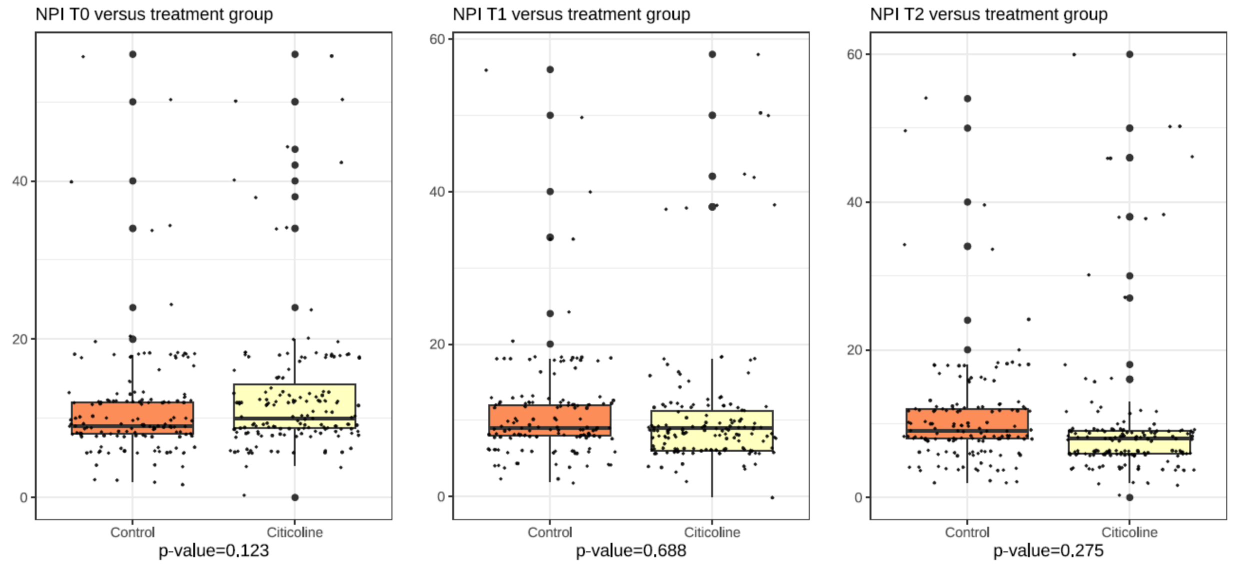

Geriatric Depression Scale (GDS)

Table 5. Summary statistics of GDS with changes from baseline at each visit.

	**Control, mean (SD)**		**Citicoline, mean (SD)**		**Total, mean (SD)**	
	**Obs. value**	**Change**	**Obs. value**	**Change**	**Obs. value**	**Change**
Baseline	2.76 (1.34)		2.55 (1.33)		2.65 (1.34)	
6 months	2.71 (1.33)	-0.04 (0.43)	2.12 (1.33)	-0.42 (0.92)	2.41 (1.36)	-0.24 (0.75)
12 months	2.6 (1.36)	-0.15 (0.76)	1.84 (1.45)	-0.71 (0.99)	2.21 (1.46)	-0.44 (0.93)

As we can see from Supplementary Table 5, treatment, time, and the interaction between treatment and time have a significant effect in explaining the GDS variable.

Figure 5. Two-way ANOVA with repeated measures over time (GDS versus time and treatment). At each time point (indicated in the plot with T0, baseline, T1, 6 months and T2, 12 months), we found a significant difference in mean between the two groups of treatment (t-test with a 5% significance level).

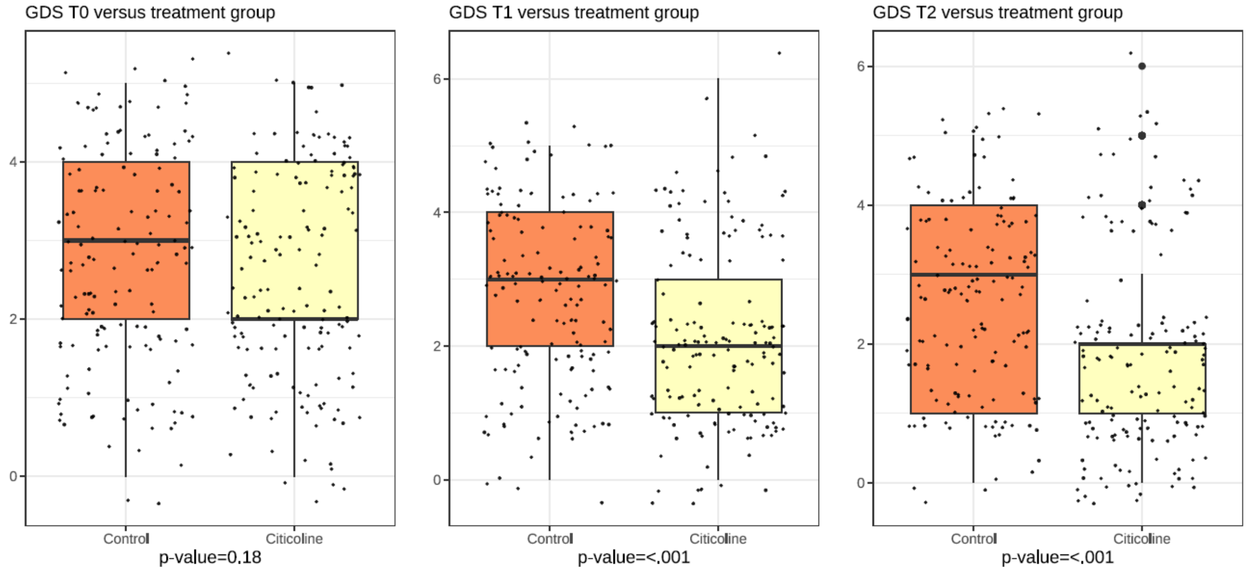

Supplement: Supplementary Material [file jad-100-jad240497-s001.pdf]
